# Endovascular repair of aortic perforation and type B aortic dissection after axillary intra-aortic balloon pump insertion

**DOI:** 10.1016/j.jvscit.2021.06.008

**Published:** 2021-08-27

**Authors:** Kelly Twohig, Ashley J. Williamson, Ross Milner

**Affiliations:** a Pritzker School of Medicine, University of Chicago, Chicago, Ill; b Department of Surgery, University of Chicago, Chicago, Ill; c Department of Vascular Surgery, University of Chicago, Chicago, Ill

**Keywords:** Dissection, Endovascular, Aortic perforation

## Abstract

We present the successful endovascular repair of an iatrogenic aortic dissection in a 57-year-old woman with decompensated heart failure. An intra-aortic balloon pump was inserted in the patient via a percutaneous axillary approach for circulatory support. Six days later, she developed symptoms of abdominal pain and lower extremity malperfusion. Computed tomography angiography demonstrated a type B aortic dissection with associated retroperitoneal hematoma secondary to aortic perforation. The patient underwent emergent endovascular aortic repair and intra-aortic balloon pump removal with return of lower extremity perfusion. She recovered well and underwent heart and kidney transplant less than 2 months postoperatively.

Stanford type B aortic dissections have been reported to occur after percutaneous femoral intra-aortic balloon pump insertion.^1^ To the best of our knowledge, this complication has not been seen in conjunction with an aortic perforation from a percutaneous axillary artery approach. We describe successful endovascular repair of the aortic perforation and associated dissection, with reperfusion of the lower extremities.

## Case

A 57-year-old woman with a history of heart failure (ejection fraction 37%), paroxysmal atrial fibrillation, mitral and tricuspid regurgitation, chronic kidney disease, and non-Hodgkin's lymphoma (status post chemotherapy and mantle radiation) was admitted for combined heart and kidney transplant evaluation. At the time of admission, the patient endorsed worsening shortness of breath on exertion and a 20-pound weight gain over 1 month. During evaluation, a percutaneous axillary intra-aortic balloon pump (IABP) was determined to be an appropriate option for circulatory support.

**Fig 1:**
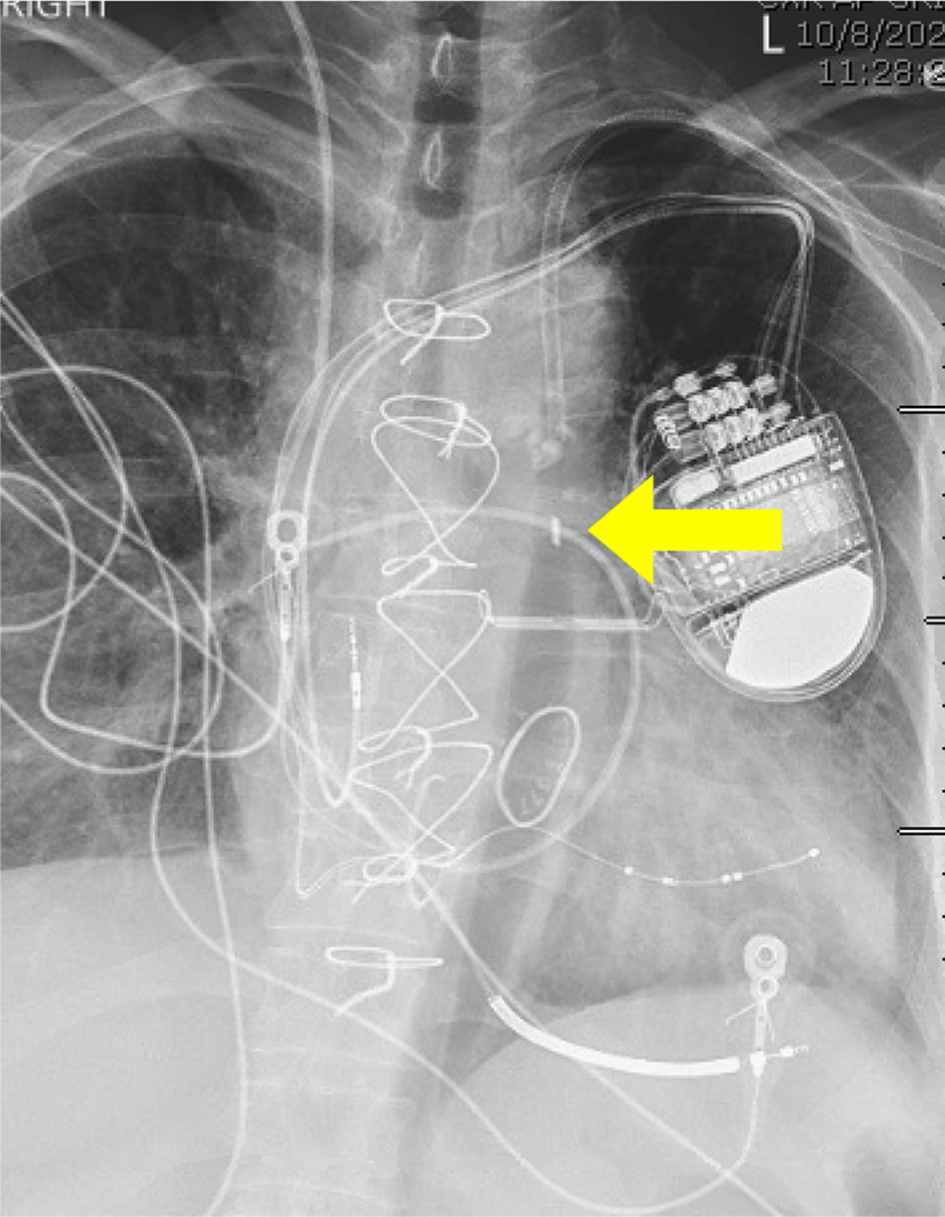
Chest x-ray after axillary intra-aortic balloon pump (IABP) placement. The cranial IABP radiopaque marker is seen in the descending aorta at the level of the left mainstem bronchus (*yellow arrow*).

**Fig 2:**
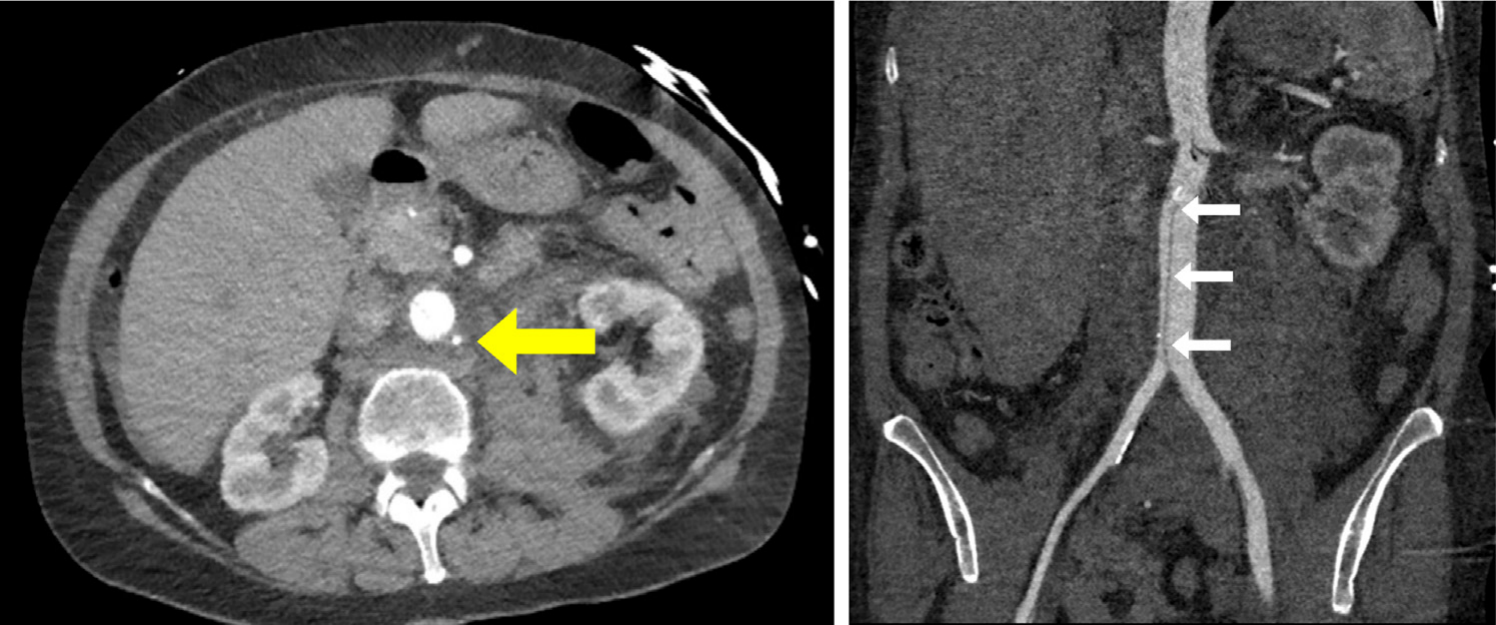
Axial computed tomography (CT) demonstrating the inferior aspect of the intra-aortic balloon pump (IABP) terminating external to the aorta (*yellow arrow*) and coronal multiplanar reconstruction CT demonstrating aortic dissection extending from the infrarenal aorta to the left external iliac arteries (*white arrows*).

On hospital day 9, the patient was taken to the operating room and the left axillary artery was accessed under direct ultrasound guidance. An IABP was advanced and positioned so that the proximal marker was just below the lower border of the left mainstem bronchus. The patient tolerated the procedure well, and good augmentation of the pump was noted. Daily chest x-rays confirmed placement of the IABP in the proximal-to mid-descending aorta (Fig 1). During the early morning of hospital day 15, the patient began to experience cramping lower abdominal pain. Simultaneously, the IABP was noted to no longer be augmenting. Computed tomography angiography (CTA) demonstrated a perforated infrarenal aorta with an associated aortic dissection extending from the tip of the balloon pump into the left external iliac artery. There was an associated retroperitoneal hematoma measuring 12.7 × 4.9 cm. The distal tip of the IABP appeared to terminate external to the aorta (Fig 2). The patient's right dorsalis pedis and posterior tibial pulses, although previously palpable, were now noted to have only Doppler signals. Her left lower extremity also demonstrated diminished flow with 1+ dorsalis pedis pulse and posterior tibial pulse only with a Doppler signal. The patient was taken emergently to the operating room with vascular surgery and cardiothoracic surgery.

The steps of this procedure are summarized in the Table. Under general anesthesia, ultrasound-guided percutaneous right common femoral artery access was obtained. Wire access was then gained adjacent to the IABP. The IABP was removed via the subclavian access site with a 23 mm aortic cuff in place from the femoral approach and ready to deploy (WL Gore & Associates, Flagstaff, Ariz). This specific model of aortic cuff was selected in light of the ability to rapidly deploy the cuff when the IABP was removed. A total of two cuffs were placed to seal the perforation and assist with treating the dissection.

**Table:** Abbreviated description of operation

1. R common femoral artery (CFA) access obtained via ultrasound guidance
2. Parallel wire access obtained through IABP sheath via the L subclavian artery
3. Aortic cuff advanced via the R CFA
4. IABP removed via the L subclavian artery
5. Total of two aortic cuffs deployed via the R CFA
6. L superficial femoral artery access obtained via the L subclavian artery
7. Total of three balloon-expandable Viahbahn stents deployed into the R and L common iliac arteries
8. Total of two self-expanding Protégé stents deployed into the L external iliac artery

*IABP,* Intra-aortic balloon pump; *L,* left; *R,* right.

**Fig 3:**
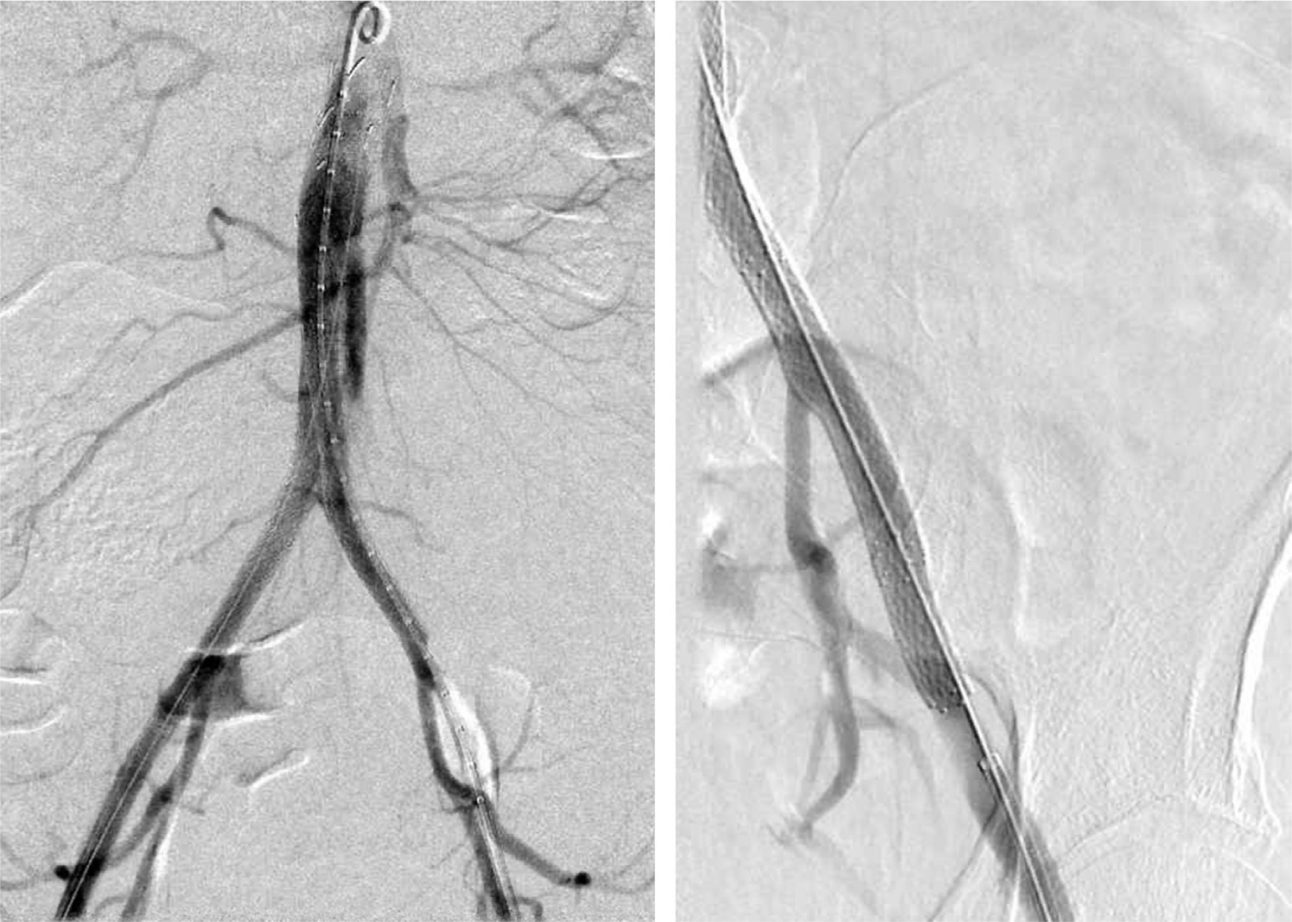
Intraoperative C-arm fluoroscopy showing two aortic cuffs and kissing iliac stents in the infrarenal aorta, as well as two Viabahn stents in the right common iliac artery and one in the left common iliac artery (left). There is good perfusion of the hypogastric arteries bilaterally; however, the true lumen of the left external iliac artery is still significantly compressed. After placement of two Protégé stents, there was resolution of the dissection and excellent flow through the left external iliac artery (right).

Percutaneous access was attempted via the left femoral artery, but the wire did not advance easily due to the dissection. Access was gained from the left subclavian artery sheath after the IABP was removed and into the left superficial femoral artery. This technique allowed for placement of the necessary iliac stents to treat the malperfusion. The right external iliac artery was thoughtfully preserved for future kidney transplantation. Contrast arteriography revealed resolution of the dissection and excellent flow throughout all of the aortic and iliac stents (Fig 3). The patient developed palpable pedal pulses bilaterally in the operating room. She was extubated and returned to the intensive care unit.

CTA completed on postoperative day 9 demonstrated patency of all stents, a stable retroperitoneal hematoma, and no evidence of extravasation. The patient was discharged home on hospital day 27.

**Fig 4:**
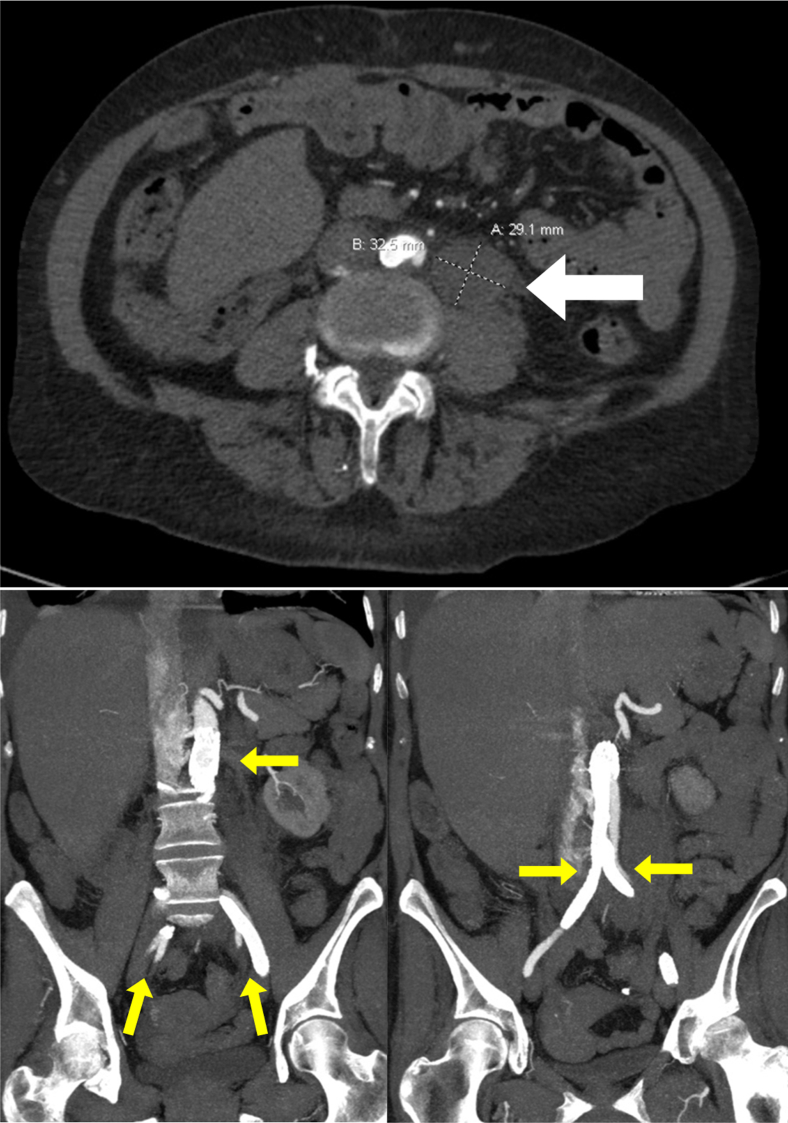
Computed tomography (CT) scan taken 1 month postoperatively demonstrating a reduction in size of a retroperitoneal hematoma (*white arrow*), as well as good perfusion through the infrarenal aorta and the bilateral common, internal, and external iliac arteries (*yellow arrows*).

She was re-admitted to our hospital 2 weeks after discharge with worsening dyspnea on exertion and a 10-pound weight gain. Repeat CTA performed 1 month after her procedure again demonstrated patency of all stents and a decrease in size of the retroperitoneal hematoma (Fig 4). The patient ultimately underwent a heart-kidney transplant on hospital day 18. Sadly, she suffered a superior vena cava (SVC) rupture during repair of an SVC stenosis 3 days after transplant. She passed away less than a month later. The patient did provide consent for publication of this case report and images.

## Discussion

Our colleagues in cardiothoracic surgery opted to place an axillary IABP in this patient. Although traditional femoral IABPs are straightforward to insert, our institution commonly places axillary IABPs in select patients due to improved mobility after the procedure. Patients can ambulate with axillary IABPs, which offers a significant advantage in recovery. Cardiac rehabilitation and improved patient mobility has become a cornerstone in the management of heart failure, as exercise training has been shown to reduce hospitalizations and mortality.^2^

Aortic dissection or perforation is a rare complication of IABP insertion, with a reported incidence of approximately 1%.^1^ Our institution published a retrospective analysis of 133 patients who underwent axillary IABP support as a bridge to transplantation from July 2009 to April 2019.^3^ Of these patients, only one (0.8%) had an acute Stanford type A dissection and required urgent surgery.^3^ No patients were reported to have Stanford type B dissections. Our colleagues previously postulated that an axillary approach may have a higher risk of type A aortic dissection than a femoral approach due to the anatomic proximity of the axillary artery to the ascending aorta. However, this case represents type B dissection as a potential, as well as serious, complication of axillary IABP insertion.

Upon literature review, few case reports have described type B aortic dissections as a complication of femoral IABP insertion.^4^, ^5^, ^6^ In two of these cases, intraoperative transesophageal echocardiograms (TEEs) revealed iatrogenic type B aortic dissections with some portion of the IABPs in the false lumens, and the catheters were immediately removed.^5^^,^^6^ These case reports demonstrate the clinical utility of intraoperative TEEs in early recognition of a potentially devastating complication. In the majority of these cases, the IABP appeared to function normally despite underlying aortic dissection. Of note, TEE was not used during IABP insertion in our patient. Postprocedural x-rays may confirm proper positioning of the IABP but are inadequate in diagnosis of this complication.

It is not entirely clear how, and when, the IABP eroded through the aorta in this patient. Insertion in this case was uncomplicated, and the IABP was correctly positioned in follow-up x-rays. One possibility is that the tip of the catheter was kinked upon insertion in an anteroposterior direction and thus was not visualized in chest radiographs. Another factor related to perforation may involve fragility of the aortic wall. Our patient went on to suffer a rupture of the SVC after heart transplant. It is possible that this patient with several comorbidities had some degree of fragility of the vessel walls. However, histopathological studies of the aorta/SVC were not performed, so any vasculopathy could not be confirmed.

This case also demonstrates the safety and efficacy of endovascular repair of aortic rupture and dissection after removal of the IABP device. The patient recovered well and was stable enough to undergo heart and kidney transplant less than 2 months postoperatively with CTA demonstrating patency of all stents.

## Conclusions

In conclusion, we present a unique case of iatrogenic aortic perforation and type B dissection after axillary IABP insertion. Although a serious complication, this can be successfully and safely repaired via an endovascular approach and still allow necessary future treatments.
